# Adventure Behavior Seeking Scale

**DOI:** 10.3390/bs7020035

**Published:** 2017-05-27

**Authors:** Piotr Próchniak

**Affiliations:** Department of Psychology, Pomeranian University, Westerplatte 64, Slupsk 76-200, Poland; piotrprochniak@wp.pl

**Keywords:** sensation seeking, adventure behavior

## Abstract

This article presents a new tool—the Adventure Behavior Seeking Scale (ABSS). The Adventure Behavior Seeking Scale was developed to assess individuals’ highly stimulating behaviors in natural environments. An exploratory factor analysis was conducted with 466 participants and resulted in one factor. The internal consistency was 0.80. A confirmatory factor analysis was performed using another sample of 406 participants, and results verified the one-factor structure. The findings indicate that people with a lot of experience in outdoor adventure have a higher score on the ABSS scale than control groups without such experience. The results also suggest that the 8-item ABSS scores were highly related to sensation seeking. The author discusses findings in regard to the ABSS as an instrument to measure outdoor adventure. However, further studies need to be carried out in other sample groups to further validate the scale.

## 1. Introduction

Adventure behavior is free-time activity that occurs outdoors with inherent elements of risk, typically taking place within natural environments [1,2]. This activity is gaining in popularity. More and more people are taking up mountaineering, canoeing/kayaking, diving, or cross-country running [3,4,5].

The positive consequences of adventure behavior are multifaceted: improving psychological and social well-being, feeling healthy, socialization, risk management, taking responsibility for oneself and others, learning and improving skills, feeling happier and relaxed, or deeply experiencing nature [6,7,8].

Adventure behavior also has multidimensional high risks associated with the real possibility of serious injury or even death. These risks are the “core” of outdoor adventure [9,10]. Perceived risk in adventure behavior is subjective and varies from person to person [3]. Therefore, situations in natural environments that present a high level of risk for one person may present only a low risk for another [3].

There are three serious environmental risks that directly impact adventure behavior in a wilderness situation: climate and weather, gravitation, and lack of oxygen [11,12]. For instance, terrains, landforms, snowstorm, avalanches, cold, and heavy wind can present risks to mountain expedition tourists. Scuba divers are confronted with the power of water and high barometric pressure. Skydivers fight against the forces of gravitation. These risks can be a cause of extreme discomfort leading to injury, illness, or even death. Therefore, adventure behavior requires great endurance, perseverance, overcoming one’s concerns, or confronting one’s fear. Practitioners of adventure behavior must be highly skilled and fit [13].

Various theories have been created to study adventure behaviors of individuals in natural environments and to predict their involvement. Zuckerman’s theory [14] posits that people who undertake risky outdoor behaviors are sensation seekers. To them, untamed nature is a place for experiencing adventure. They perceive natural environments as a source of stimulation.

For Priest [15], adventure behavior is an experiential method for learning new skills, which requires the use of all senses and cognitive processes. Adventure behavior is based upon personal experience, and it is a matter of relationships involving individuals and the constraints of nature. Similar notions can be found in Csikszentmihaly’s theory of *flow*. Csikszentmihalyi [16] put forward a theory highlighting the synergy between personal skills and the challenges presented to the individual by natural environments. An insufficient skill set or an overly demanding challenge means that the adventure activity in question will not be undertaken. In the theory of Bandura, participation in outdoor adventure is determined by the perceived level of one’s competences, i.e., self-efficacy [17]. Self-efficacy is positively connected with the frequency and difficulty of doing outdoor activities, regardless of their forms. An increasing sense of self-efficacy motivates one to engage in voluntary outdoor activities in threatening natural environments [18,19,20,21].

Surprisingly however, the research into this has very often focused on theoretical considerations; in the literature, there have been few instruments that measure this issue. To fill this gap, the goal of this study is the development of the Adventure Behavior Seeking Scale based on the sensation-seeking theory proposed by Zuckerman [14].

### Sensation Seeking

The most widely known research on risky behavior within natural environments is Marvin Zuckerman’s work on the personality trait called sensation seeking [14]. The sensation-seeking trait is defined *as the seeking varied, novel, complex, and intense sensations and experiences, and the willingness to take physical, social, legal and financial risks for the sake of such experiences* [14] (p. 27). Actually, the explanation for sensation seeking is based on a model influenced by biological, psycho-physiological, and social factors. These factors determine specific preferences and behaviors [22,23,24]. The sensation-seeking trait has negative and positive consequences [25]. It is related to using drugs [26], alcohol [27,28], or engaging in risky sexual behavior [29]. The sensation-seeking trait is also related to participating in risky behaviors in close contact with natural environments [30,31,32,33,34,35,36,37,38,39,40].

The research conducted in the area of sensation seeking has used the Sensation Seeking Scale. It is considered a valuable tool in the assessment of the need for stimulating experiences in many areas, including the broadly understood psychology of outdoor activity.

Work on the questionnaire began in the 1960s. Initially, sensation seeking was treated as a single, general trait in the Sensation Seeking Scale (Form I and II in the scale) [41]. However, factor analyses of the statements in the questionnaire (Form I and Form II) suggested a multifactor solution as a better choice than a single, general factor [42,43].

Successive versions of the questionnaire (Form II, IV, V, VI) allow for a four-factor solution to sensation seeking: Thrill and Adventure Seeking (TAS); Experience Seeking (ES); Disinhibition (Dis); Boredom Susceptibility (BS) [44]. Individual scales differ with regard to the number of statements they include [45,46]. The SSS V, possibly the most popular version of the scale, includes 40 statements [47]. It was translated into many languages and adapted in many countries [48,49,50,51,52,53,54].

However, the measure has received criticism for its length, thereby prompting others to develop self-report alternatives. Among the new measures is the Brief Sensation Seeking Scale—the BSSS. The Brief Sensation Seeking Scale includes two items representing each aspect of sensation seeking [29].

The Sensation Seeking Scale does have other limitations, as indicated by Arnett [22]. Because of its yes/no answer checklist, it can cause some frustration, as respondents may be inclined to answer yes/no simultaneously. Furthermore, some statements concerning such activities as climbing or skydiving are strongly correlated with physical strength. The scale also includes statements which are characteristic for the Euro-American culture, which makes it difficult for people from different cultural backgrounds to understand the statements. Arnett [22] suggests his own version of the Sensation Seeking Scale, assuming a two-factor solution: novelty and intensity.

It seems that the above list of reservations made by Arnett [22] can be developed, particularly in the context of diagnosing the intensity of thrill and adventure seeking in natural environments.

Another important limitation of the scale is the fact that respondents express their declaration to participate in an activity and not real behavior (e.g., “I often wish I were a mountain climber”). The answers to the questionnaire are usually given in a safe environment and do not reflect the real context in which a given outdoor activity is performed. This is why people can declare their participation in a given activity, whereas in real and threatening environmental conditions these declarations may not be upheld.

Another significant problem with the thrill- and sensation-seeking scale concerns the predictability of the answers given by those respondents who provide themselves with new and intense sensations through participation in risky sports in close contact with natural environments. One can hardly expect a person who participates in skydiving to give a negative reply to the statement, “I would like to try parachute jumping.”

The Sensation Seeking Scale also does not include various sources of risk found in natural environments, especially those connected with weather. Individuals can seek sensations through contact with one source of risk, such as gravity, and outdoor activity in severe weather conditions may not be of interest to them. Obviously, weather conditions are the universal feature when participating in activities in close contact with natural environments [55,56].

It appears that using activities which require technical equipment (parachute, skis) in the statements is also a significant limitation of the scale. Not everyone can afford such equipment, and it is this reason, not actual lack of interest, that can determine resignation from seeking sensation in participating in specific activities in close contact with nature.

The author’s suggestion for a thrill- and adventure-seeking scale attempts to take at least some of these limitations into account. This is why the following assumptions were made with regard to constructing the scale:

- The checklist should be based on Likert’s scale.
- The scale should omit statements concerning declarations and not factual behavior.
- The scale should take into account various sources of risk within natural environments.
- The scale should not include statements which mention using technical equipment in outdoor activity. In other words, adventure seeking in natural environments should require physical and mental health only.
- The scale should include statements which can be understood by people of various cultural backgrounds.

The remarks above were included in the development of the Adventure Behavior Seeking Scale.

## 2. Construction of the Adventure Behavior Seeking Scale

The author obtained institutional review board approval for the study.

### 2.1. Method

#### 2.1.1. Participants

In order to examine the factor structure of the newly developed scale, data were collected from two separate groups of participants. The first sample consisted of 466 university students (230 women and 236 men, Mage = 23.20; SD = 6.65) recruited from a variety of courses, i.e., management (*N* = 180), social work (*N* = 48), sociology (*N* = 38), and medical emergency (*N* = 200) at an urban university in Poland. In total, 73% of the participants were from cities and 27% from villages.

The respondents in this group practiced the following outdoor activities: mountain climbing (9.50%); skiing (12.50%); snowboarding (4.80%); running (42%); orienteering (9.40%); rafting (3.20%); sailing (6.30%); windsurfing (3.40%); waterskiing (1.10%); scuba diving (4.90%); cycling (48%); skydiving (2.30%); paragliding (1.90%); horse riding (22.00%); others (27.00%), (the sum of percentages being higher than 100 because some respondents practiced more than one outdoor recreational activity). Data obtained from this sample were examined using exploratory factor analysis.

The second sample included a separate group of 406 university students (201 women and 205 men, Mage = 21.65; SD = 4.25) recruited from a variety of courses: biology (*N* = 27), mathematics (*N* = 26), pedagogy (*N* = 160), and national security (*N* = 193). In total, 68% of the participants were from cities and 32% from villages.

The respondents in this group practiced the following outdoor recreational activities: mountain climbing (12.50%); skiing (19.30%); snowboarding (4.00%); running (39%); orienteering (17.40%); cycling (49%); rafting (3.40%); sailing (5.20%); windsurfing (2.60%); waterskiing (2.60%); scuba diving (7.00%); skydiving (3.20%); paragliding (1.20%); horse riding (18.80%); others (34%), (the sum of percentages being higher than 100 because some respondents practiced more than one outdoor recreational activity).

#### 2.1.2. Procedure

The notion of designing a new scale sprang from several sources, namely, the author’s own experiences in outdoor adventure (climbing), sensation-seeking theory, literature related to outdoor recreation, and the existing scales for measuring sensation seeking.

Authors have distinguished various distinctive forms of adventure behaviors including different environmental risks. These risks vary in line with the type of adventure behavior being pursued; they may entail pitting oneself against the forces of gravitation, confrontation with the power of water, or taking on nature’s challenging landforms. Defined sets of characteristics thus also differentiate one adventure behavior from another. What is interesting, however, is that all the above-mentioned behaviors share a common feature in the challenging, and sometimes downright extreme, weather conditions under which they might well be undertaken [4]. These characteristics were the inspiration for this study. Here, adventure behavior was defined as a construct that initially was conceptualized to be comprised of three dimensions: (a) adventure behavior including confrontation with the power of water; (b) adventure behavior against the forces of gravitation; (c) adventure behavior which entails enduring challenging weather conditions [12].

A set of 18 items was generated for the new scale based on sensation seeking theory [14,29] and literature related to adventure recreation [4,8]. The statements take into account adventure behavior from the perspective of three basic sources of environmental risks: adventure behavior including water threats (six statements), e.g., “I swim far from the shore”; adventure behavior including gravity threats (six statements), e.g., “I climbed high trees”, and adventure behavior including weather threats (six statements), e.g., “I spend my leisure time in a forest even when the day is cold”.

The statements do not include declarations of willingness or inclinations towards a given activity, even a risky one. Instead of declarations regarding preferred outdoor activities, the statements concern events, which refer to specific behaviors in natural environments. Moreover, the statements describe adventure behavior in the wilderness without the necessity to use specialist equipment, such as parachutes, skis or boats.

To assess the quality of the construct, six experts were asked to use a five-point Likert-type scale (very good, good, fair, poor, very poor) to independently determine the extent to which the initial pool of eighteen items (a) reflected the definition of adventure behavior (relevancy) and (b) were clearly and simply written (clarity). Each expert had a university degree in social sciences, and each expert had personal experience in an outdoor activity (three experts were climbing instructors and three experts were sailing instructors). Items were retained if the average rating on relevancy and clarity was 4.0 or higher.

Six items were dropped because they were deemed to be a poor indicator of the construct (e.g., “I like taking walks in close contact with the environment in winter time.”).

Twelve statements qualified for further examination.

Next, the respondents—students—were approached by a researcher in different outdoor activities. The researcher provided them with a general verbal introduction to the study. They were then asked to volunteer to complete the survey. Those who agreed completed the questionnaire anonymously.

### 2.2. Results

#### 2.2.1. Exploratory Factor Analysis (EFA)

Prior to factor extraction, the Kaiser–Meyer–Olkin (KMO) measure of sampling adequacy and Bartlett’s Test of Sphericity (BTS) were applied to the data. The KMO measure was found to be 0.82, and meeting Bartlett’s Test of Sphericity reached statistical significance with χ2 (66) = 1456.00; *p* < 0.001, thus fulfilling the prerequisites for conducting the EFA.

An exploratory factor analysis of the sample of 466 individuals was performed for the twelve items. Because the item pool was small, the maximum-likelihood method of parameter estimation was chosen [5,57]. Exploratory factor analysis using the maximum-likelihood method of parameter estimation indicated a single-factor solution upon inspecting the scree plot [58]. In determining the optimal number of factors to extract, Parallel Analysis (PA) was also used. Parallel Analysis is a Monte Carlo simulation technique that aids researchers in determining the number of factors to retain in Principal Component and Exploratory Factor Analysis. This method provides an alternative to other techniques that are traditionally used for the same purpose, such as the scree plot [59]. See Table 1.

Based on these results, a one-factor solution seemed to be the best solution.

The quality of the items that composed the one-factor solution was also analyzed. Comrey and Lee [60] classified items with loadings higher than or equal to 0.71 as excellent; higher than or equal to 0.63 as very good; higher than or equal to 0.55 as good; higher than or equal to 0.43 as reasonable; higher than or equal to 0.32 as poor. Thus, as to the items’ quality, four statements loaded below 0.40 and were rejected. Eight statements were accepted for the final version of the Adventure Behavior Seeking Scale. All items loaded on this factor, ranging from 0.43 to 0.63. See Table 2.

*Cronbach’s α* for the 8-item ABSS was 0.80, and item-total correlations ranged from 0.41 to 0.61. See Table 3.

#### 2.2.2. Confirmatory Factor Analysis (CFA)

To test the factor structure of the 8-item Adventure Behavior Seeking Scale, a CFA was conducted on the second group of 406 participants. The data were fit with the one-factor measurement model. The model fit indices for the CFA were found to be as follows: χ2 (df) = 80.58, *p* = 0.01; GFI = 0.940; AGFI = 0.910; CFI = 0.915; RMSEA = 0.048. The results showed that the model with the accepted fit was the single-factor model. Cronbach’s α for the single-factor model of ABSS in this group of participants was 0.80.

## 3. ABSS and Outdoor Sports

The purpose of this study was to provide the criterion validity of the Adventure Behavior Seeking Scale. The scale functions among people with more versus less experience in outdoor adventure were analyzed, and it was hypothesized that individuals with more experience in outdoor adventure would report higher scores on the dimension of the ABSS than people with less experience in outdoor adventure.

### 3.1. Method

#### 3.1.1. Participants

The Adventure Behavior Seeking Scale was distributed to 27 mountain climbers (Mage = 26.15; SD = 7.10); 30 skydivers (Mage = 23.60; SD = 3.80), and 31 representatives of water sports, i.e., surfers (Mage = 24.70; SD = 4.39). All participants were male. The control group was comprised of 83 males, who did not participate in risky outdoor activity (Mage = 25.80; SD = 7.10). The participants from the control group participated in running in natural terrain.

#### 3.1.2. Procedure

The winter climbers were recruited from climbing clubs in Poland. All climbers had personal experience in winter expeditions on peaks in the Tatra Mountains (Europe). The skydivers group was recruited through associations or clubs in northern Poland. The scuba diving group was recruited through clubs in northern Poland. All participants in this group had personal diving experience in the Baltic Sea.

The control group was recruited during the summer period. All respondents in the control group preferred easy recreation (running), and they were not interested in adventure recreation. Prior to testing, the researcher asked individuals in the control group how much time they spend in green areas and what sort of leisure time they prefer.

The researcher informed the participants about the goals of the study and handed out the ABSS scale. The participants were asked to fill out a written consent, to carefully read directions of the scale, and to raise their hands if they had any questions. The participants filled in the questionnaire individually.

### 3.2. Results

Prior to the comparison of risk takers and controls, possible differences on the ABSS scale within groups of adventure sportsmen were tested. A one-way analysis of variance revealed a lack of significant differences among these groups of participants. Thus, in the next stage, this group was described as “adventure behavior seekers.” Table 4 presents the results of the ABSS questionnaire of people who participate in risky outdoor sports and of the control group.

Adventure behavior seekers obtained higher results in the Adventure Behavior Seeking Scale than the control group (*p* < 0.01).

## 4. ABSS and the Sensation Seeking Scale

In this study, the convergent validity was assessed by comparing the ABSS scale scores with responses to another scale that was considered to measure similar constructs; thus, possible correlations between the ABSS and the Sensation Seeking Scale (SSS IV) were checked.

### 4.1. Method

#### 4.1.1. Participants

The sample consisted of 380 university students (193 women and 187 men, Mage = 23.20; SD = 6.65) recruited from a variety of courses (management (*N* = 120); medical emergency (*N* = 140); national security (*N* = 120) at an urban university in Poland.

The respondents practiced the following outdoor activities: mountain climbing (7.40%); skiing (15.50%); snowboarding (3.40%); cycling (48%); rafting (2.20%); sailing (7.50%); windsurfing (4.40%); scuba diving (5.30%); running (44.30%); skydiving (2.40%); paragliding (1.60%); horse riding (25.00%); others (30.00%), (the sum of percentages being higher than 100 because some respondents practiced more than one outdoor recreational activity). In total, 69% of the participants were from cities and 31% were from villages.

#### 4.1.2. Procedure

The respondents were informed of the aim of the study. Prior to testing, the researcher asked individuals what sort of activities they practice in natural environments. Each respondent was given a set of questionnaires. The respondents were asked to complete the questionnaires carefully. Participation was voluntary and anonymous.

#### 4.1.3. Measure

##### Sensation Seeking Scale IV

The Polish version of the SSS IV consists of 68 items comprising six scales: general tendency towards sensation seeking (G), Thrill and Adventure Seeking (TAS), Experience Seeking (ES), Disinhibition (Dis), Boredom Susceptibility (BS), and Intellectual Stimulation Requirement (I) [61].

In this study, five scales of the SSS IV were used: Thrill and Adventure Seeking (Cronbach’s α = 0.79), Experience Seeking, (Cronbach’s α = 0.75), Disinhibition (Cronbach’s α = 0.73), Boredom Susceptibility (Cronbach’s α = 0.70), and General Sensation Seeking (Cronbach’s α = 0.82).

### 4.2. Results

The results are presented in Table 5.

The Adventure Behavior Seeking Scale correlates positively with the following scales: Thrill and Adventure Seeking, Experience Seeking, Disinhibition, Boredom Susceptibility, and General Sensation Seeking (*p* < 0.01).

## 5. General Discussion

The purpose of these studies was to develop and validate the ABSS to assess individuals’ adventure behavior within natural environments. The validity of ABSS was tested using exploratory and confirmatory factor analysis. The results indicate that despite distinguishing various sources of risk in natural environments, the statements did not become arranged in several separate factors. One factor encompassing varied forms of threats in natural environments was the best solution for the Adventure Behavior Seeking Scale. In this respect, the single-factor solution is similar to the traditional Thrill and Adventure Seeking Scale.

The internal consistency of the scale is sufficient to conclude that items are good indicators of the adventure behavior seeking construct. The item-total correlations of individual statements in the questionnaire range from 0.41 and 0.61. The scale differentiated the respondents with regard to their need for exploration of wilderness.

The Adventure Behavior Seeking Scale correlates positively with the SSS IV scale. Significant positive correlations of the ABSS scale with the SSS IV scale provided evidence of convergent validity and also indicated that adventure behavior may be considered a component of a broader sensation-seeking construct.

Positive correlations were found between the ABSS scale with both sensation-seeking measures. The ABSS scale correlated more strongly with the SSS-TAS scale than with the SSS-ES, SSS-DIS, or SSS-BS scales. This result suggests that the ABSS scale is rather more closely associated with seeking behaviors that involve outdoor adventure than seeking experiences through mind and senses, disinhibition, or boredom.

It appears that the advantage of the scale is that it diagnoses specific forms of behavior displayed by the respondents in natural environments rather than relying on their declarations concerning the choice of risky activity. It does not evoke specific disciplines or forms of outdoor activity, which means that the problem of tautological statements, as in the case of the TAS scale, has been eliminated.

The Thrill and Adventure Seeking Scale suggested by Zuckerman includes statements mentioning particular outdoor sports (e.g., skydiving, mountain climbing, and sailing). The new adventure-seeking scale makes it possible to avoid concentrating on specific outdoor sports, which can promote more precise comparisons between people preferring various forms of activity in natural environments.

The ABSS scale does not include statements mentioning the use of technical equipment in outdoor activity. Also, a four-point Likert format was used for the ABSS scale with the intention of offering more response choices. It seems that this is the advantage of the ABSS scale.

The above points suggest that the ABSS scale can be used as an alternative to the Thrill and Adventure Seeking scale [14].

This scale may be useful for research when researchers are pressed for time because the ABSS scale consists of only eight items. Gosling and his coworkers accept the use of shorter scales because these instruments reduce respondent fatigue and the *oft-expressed frustration of answering the same question again and again* [62] (p. 524).

The findings of the study can also have significant managerial benefits. Tourist counselors can use this measure when working with clients. If counselors understand the level of adventure behavior seeking of tourists, they will be in a better position to assist tourists in overcoming challenges by selecting behaviors with the appropriate level of challenge. In this way, the adventure-seeking tourists will be satisfied when visiting a destination that reflects their preferences.

## 6. Limitations and Future Directions

An important limitation of the present study is that the participants were mainly university students. This fact limits the generalization of the results. In further research, it will be important to assess not only students, but other groups of people as well. Further studies should also assess the role of gender. The current study did not measure the role of gender for adventure behavior seeking.

The data were collected in Poland. The scale includes statements which are characteristic for the Polish culture, which makes it difficult for people from different cultural backgrounds to understand the statements. Therefore, caution must be taken when this measurement is applied to other countries.

Results obtained in the ABSS scale from people who perform outdoor sports indicate that the scale differentiates people on the basis of their needs within the scope of thrill and adventure seeking in contact with natural environments. The question remains to what extent the observed differences are the result of the participants’ physical strength, and to what extent they are a consequence of the varied level of sensation seeking among the respondents. This issue has not been resolved, and this can be considered a limitation of the scale.

Future studies should show the relationship between adventure seeking in natural environments and the basic models of personality traits, i.e., the Big Five and the Alternative Five Factor Model [63,64]. Previous studies indicate that thrill seeking correlates with openness to experience and with lower conscientiousness [14]. Do similar dependencies exist for the ABSS scale?

It is easier to predict the relationship between adventure-behavior seeking and the alternative five-factor model. Adventure seeking is probably connected to Impulsive Sensation Seeking. The question of whether adventure seeking is related to aggression-hostility, as indicated by Arnett [22], remains open.

Future studies can also focus on the potential connections between the ABSS and the scale suggested by Arnett [22]. In this context, it is interesting which (if any) dimensions of the Novelty–Intensity Scale correlate with the ABSS.

Also, more research is needed to continue to evaluate the complex relationship between the Adventure Behavior Seeking Scale and other physical and psychosocial variables in order to fully understand the determinants of effective behavior in wilderness. For example, future studies could focus on the potential connections between the ABSS and the Self-Efficacy Scale [65]. Moreover, future research could analyze the consequences of adventure behaviors for mental and physical well-being [66,67]. Can adventure behaviors contribute to optimism, vitality, positive self-esteem, physical health, and overall life satisfaction?

Future studies using the ABSS scale could also concern the perception of outdoor risk, depending on the intensity of sensation seeking in natural environments. Previous studies indicate that people who display a high intensity of sensation seeking underestimate the risks connected with doing outdoor sports [68]. Do similar dependencies exist for the ABSS scale?

## 7. Conclusions

Measuring constructs that can predict behaviors across a variety of areas is becoming important. Surprisingly, in the literature there have been few instruments that measure behaviors within natural environments. To fill this gap, the goal of this study was development of the Adventure Behavior Seeking Scale. The results obtained make it possible to posit that the Adventure Behavior Seeking Scale is a valid and reliable diagnostic tool. It is hoped that ABSS will contribute to the identification of risky behaviors in a wilderness areas.

## Figures and Tables

**Table 1:** Parallel Analysis for the Adventure Behavior Seeking Scale

Variable	Note: *N* = 466		
	PCA	PA	Differences
1	4.25	1.26	2.99
2	0.82	1.19	−0.37
3	0.69	1.14	−0.45
4	0.65	1.09	−0.44
5	0.54	1.05	−0.51
6	0.51	1.01	−0.47
7	0.45	0.97	−0.52
8	0.41	0.93	−0.52
9	0.39	0.89	−0.50
10	0.36	0.85	−0.49
11	0.35	0.81	−0.46
12	0.34	0.76	−0.42

PCA: Principal Component Analysis; PA: Parallel Analysis.

**Table 2:** Exploratory Factor Analysis of the Adventure Behavior Seeking Scale

No.	Item (by Content Domain)	M	SD	Loadings
	*Adventure behavior including water threats*			
1	I swim far from the shore	2.08	1.14	0.62
3	I try to check how long I can stay underwater	2.19	1.05	0.63
5	I jump off steep slopes into water	1.49	0.95	0.62
	*Adventure behavior including gravity threats*			
4	I climbed high trees	2.01	1.07	0.61
6	I go for a hike	1.99	1.01	0.63
	*Adventure behavior including weather threats*			
2	I go in for outdoor recreations even when it’s cold or there’s a strong wind	1.70	0.99	0.43
7	Mud and dust don’t put me off trekking	2.19	1.05	0.44
8	I jump into cold water without preparation	1.51	0.84	0.51

**Table 3:** Reliability and Item-Total Correlations for the Adventure Behavior Seeking Scale

*The ABSS Items*	*Scale Mean If Item Deleted*	*Scale Variance If Item Deleted*	*Corrected Item-Total Correlation*	*Cronbach’s* α *If Item Deleted*
1	13.35	21.29	0.54	0.77
2	13.62	23.27	0.44	0.78
3	13.12	21.16	0.53	0.77
4	13.36	21.48	0.52	0.77
5	13.51	21.72	0.61	0.77
6	13.07	22.40	0.52	0.79
7	13.85	23.76	0.41	0.78
8	13.81	22.35	0.42	0.78
Cronbach’s α				0.80

**Table 4:** The Adventure Behavior Seeking Scale and Outdoor Sports

Variable	Adventure Behavior Seekers (*N* = 84)		Controls (*N* = 83)		t	Cohen’s d
	M	SD	M	SD		
ABSS	2.65	0.64	1.97	0.74	6.32 *	0.98

* *p* < 0.001.

**Table 5:** The Adventure Behavior Seeking Scale and the Sensation Seeking Scale IV.

SSS IV	ABSS
Thrill and Adventure Seeking	0.61 *
Experience Seeking	0.47 *
Disinhibition	0.45 *
Boredom Susceptibility	0.45 *
General Sensation Seeking	0.65 *

* *p* < 0.01.
